# Gamma-Aminobutyric Acid (GABA) Avoids Deterioration of Transport Water Quality, Regulates Plasma Biochemical Indices, Energy Metabolism, and Antioxidant Capacity of Tawny Puffer (*Takifugui flavidus*) under Transport Stress

**DOI:** 10.3390/biology13070474

**Published:** 2024-06-26

**Authors:** Xiaowen Yu, Wenjie Hou, Lixia Xiao

**Affiliations:** 1 Shanghai Fisheries Research Institute, Shanghai 200433, China; xywinni@163.com; 2 Qidong Fishery Technology Promotion Station, Qidong 226299, China; 13962903338@qidong.gov.cn

**Keywords:** *Takifugu flavidus*, GABA, transport stress, energy metabolism, antioxidant capacity

## Abstract

**Simple Summary:**

Live fish transport often induces stress, while gamma-aminobutyric acid (GABA) is considered to exhibit anti-stress effects. This study aims to explore the effects of GABA on alleviating the transport stress of tawny puffer (*Takifugu flavidus*) by detecting water quality parameters, plasma biochemical indices, energy metabolism indicators, and antioxidant indexes. The results indicated that 50 and 150 mg/L GABA treatments exert anti-stress effects significantly on the tawny puffer, which provides a scientific basis for the development of GABA as an effective additive for aquatic animals’ live transport.

**Abstract:**

Live fish transportation is crucial for managing aquaculture but can pose health risks to fish due to stressors encountered during transportation. Gamma-aminobutyric acid (GABA) is an inhibitory neurotransmitter that plays a crucial role in the central nervous system and is considered to exhibit anti-stress effects. This study aims to investigate the effects of GABA on the transport water quality, plasma biochemical indices, energy metabolism, and antioxidant capacity of tawny puffer (*Takifugu flavidus*) under transport stress. Tawny puffer were pretreated by immersing in aquariums containing GABA (final concentrations at 0, 5, 50, and 150 mg/L) seawater for 3 days; then, simulated transport was conducted using oxygen-filled polyethylene bags containing the same concentration of GABA seawater as the pretreatment period. Water samples, plasma, and liver were collected after 0, 6, and 12 h of transport. The results revealed that with the prolongation of transportation time, the control group’s water quality deteriorated, stress-related plasma biochemical indices increased, glycolytic substrate contents decreased, glycolytic enzyme activities and product contents increased, and aerobic metabolic enzyme activities exhibited initial increases followed by declines, ATPase activities decreased, antioxidant enzyme activities decreased, and the lipid peroxidation marker contents increased. It is noteworthy that GABA treatment could avoid water quality deterioration during transportation, inhibit an elevation in stress-related biochemical indicators, regulate energy metabolism, and reduce oxidative damage in tawny puffer, especially at 50 and 150 mg/L concentrations. In summary, GABA treatment can effectively alleviate the transport stress of tawny puffer.

## 1. Introduction

In aquaculture management, the transport of live fish is a routine operation firmly associated with economic benefits, which is essential for fish sales, stock enhancement, etc. [1]. One of the ordinary and economical methods for live fish transport is oxygen-filled and sealed transportation by polyethylene bags. However, water quality in polybags usually deteriorates with the length of the transport period [2]. The most basic parameters for determining water quality include pH, dissolved oxygen (DO), and total ammonia nitrogen (TAN) [3]. Hypoxia, pH decrease, TAN increase, shakes, and collisions are common stressors in live fish transport, and multiple stressors can trigger stress responses in fish [4].

Stress is a non-specific physiological response to environmental stimuli and a defense mechanism [5]. Appropriate stressors help organisms adapt to ecological changes rapidly, while excessive stressors cause serious health hazards [6]. Typically, stress responses are categorized into primary stress responses (short-term), secondary stress responses, and tertiary stress responses (long-term) [7,8]. The primary stress response is a neuroendocrine reaction. Fish have two stress hormone reaction systems: one is the sympathetic–chromaffin (SC) axis, and the other is the hypothalamic–pituitary–inter-renal (HPI) axis [9]; the stimulation of the former can induce the secretion of stored catecholamine hormones in abundant quantities from chromaffin cells in tissues (primarily the head kidney), resulting in a notable elevation in epinephrine (EPI) or norepinephrine levels in the blood; the latter is stimulated through a cascade of corticotropin-releasing factor (CRF), adrenocorticotropic hormone (ACTH), and corticosteroids, ultimately leading to a significant increase in cortisol (COR) content in the blood. The secondary stress response encompasses a range of physiological alterations induced by the primary stress response, such as modifications in energy metabolism. The tertiary stress response refers to the individual or group-level changes caused by stress. It is worth noting that stress-related metabolic activities lead to the production of reactive oxygen species (ROS), which cause organism oxidative damage when an antioxidant defense system does not counter excessive ROS [10]. The antioxidant enzyme activity in liver tissue is used as a stress indicator of fish after transport in many studies [11].

Overall, alleviating fish transport stress is essential for improving the transportation survival rate, maintaining fish health and welfare, and ensuring fish meat quality [12]. Prolonged transportation stress causes a reduction in the physiology of fish, leading to increased mortality rates and economic losses, threatening the sustainability of the aquaculture industry.

Various methods have been shown to alleviate fish transport stress, including optimizing transportation facilities [13], adding anesthetics [14], etc. However, these methods have limitations, such as high equipment costs and drug residues. Therefore, developing efficient and convenient anti-stress additives is imperative. Gamma-aminobutyric acid (GABA) is a non-protein amino acid found widely in animals, plants, and microorganisms [15]. In recent years, the function of GABA in vertebrates has become clearer with in-depth research. GABA has antioxidant properties, helps in immunomodulation, and promotes the growth of organisms [16,17]. As an inhibitory neurotransmitter, GABA plays a vital role in the central nervous system, as it has calming, anxiolytic, anti-stress, and anti-hypoxic protective effects [18]. In research on aquatic animals, dietary GABA supplementation could enhance the hypoxia tolerance of juvenile Chinese mitten crab (*Eriocheir sinensis*) by regulating respiratory metabolism and alleviating neural excitotoxicity [19]. The studies of Yan et al. [20] have demonstrated that GABA addition in transport water improved the anti-stress ability of Crucian carp (*Carassius auratus*) by inhibiting metabolism and reducing COR contents; the studies of Wu et al. [21] showed that GABA addition in transport water improved the stress tolerance of Koi carp (*Cyprinus carpio*) by effectively suppressing the increase in EPI, COR, and GLU contents of the blood. This indicates that GABA can alleviate transport stress in several freshwater aquatic animals. However, the anti-stress effect of GABA on marine fish’s live transport is unclear.

Tawny puffer (*Takifugu flavidus*) is a temperate bottom fish species mainly distributed in the coastal waters of the East China Sea, Yellow Sea, and Bohai Bay. Tawny puffer has very high edible and medicinal values, thus becoming a potential economic aquaculture species [22]. According to the China Fishery Statistical Yearbook, the yield of farmed tawny puffer stood at 16,626 tons in 2022, jumping by 8.02% from the previous year. With the increasing exploitation of oceans globally, stock enhancement is becoming an important way to protect fishery resources [23], and the tawny puffer is one of the key species. During the stock enhancement and sale process, tawny puffer cultivated on hatcheries that achieve certain stages (typically juveniles) must be transported alive to the sea area, while tawny puffer juveniles are susceptible to experiencing stress during transportation; in addition, tawny puffer have hard teeth and will bite each other when kept in a high density, which potentially causes additional morbidity and mortality. Together with the high economic value of the tawny puffer, its transportation failure may lead to severe losses; thus, it is urgent and necessary to explore a method of alleviating its transport stress. In view of the above, the present study takes tawny puffer juveniles as the subject and is dedicated to exploring the effect of GABA in alleviating transport stress by detecting water quality parameters, plasma biochemical indices, energy metabolism indicators, and antioxidant indexes, which will establish a foundation for further investigations into the functions and mechanisms of GABA and provide a scientific basis for utilizing GABA as an effective additive in the live transport of aquatic animals.

## 2. Materials and Methods

### 2.1. Experimental Animals

Tawny puffer juveniles (14.91 ± 2.84 g) were obtained from the Qidong Research Base of Shanghai Fisheries Research Institute. The experimental tawny puffer were acclimatized for 3 days under laboratory conditions before the formal experiment. During the ecological adaptation process, filtered and aerated natural seawater was supplied and changed daily. The fish were fed a commercial diet (Grobest, Taiwan, China) twice daily. The water temperature was maintained at 25.35 ± 0.56 °C, the pH at 7.51 ± 0.09, and the DO level at 7.62 ± 0.57 mg/L. All laboratory animals were handled following the guidelines of the Care and Use of Laboratory Animals at the Shanghai Fisheries Research Institute.

### 2.2. GABA Pretreatment Experiment

The GABA (≥99%) used in this study was sourced from Sigma Co., Ltd. (Shanghai China). A total of 216 experimental tawny puffer (healthy and active) were immersed for 3 days in aquariums (containing 4.5 L seawater) with final concentrations of GABA at 0, 5, 50, and 150 mg/L [20,21]. There were three replicates in each group, and each replicate contained 18 fish. Tawny puffer were fed to satiation twice daily during the GABA treatment period. All experimental seawater was changed daily, and the same concentration of GABA was supplied. Other experiment conditions were consistent with the adaptation period. Tawny puffer were fasted for 24 h before the transport experiment.

### 2.3. Simulative Transportation Experiment

**Figure 1:**
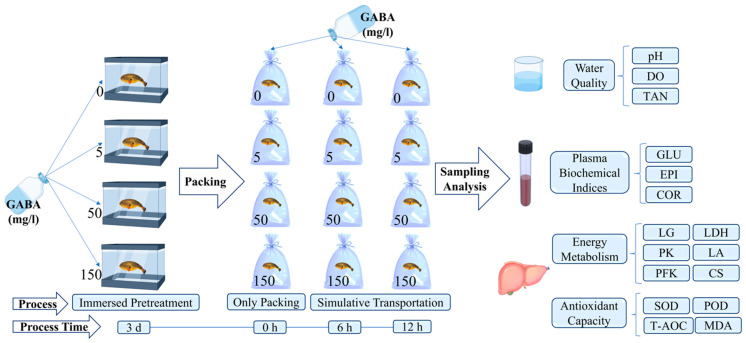
Schematic representation of the experimental design of this study.

The experiment was designed to simulate live fish transport using polybags containing four final concentrations of GABA (0, 5, 50, 150 mg/L) seawater at three transport times (0, 6, and 12 h). The 0 mg/L GABA treatment group was used as the control group. The time of 0 h transport means only packing, but no transportation operation had been conducted. Polybags of four GABA treatment groups contained 1.5 L of filtered natural seawater, and three replicates were set at each transport point. Tawny puffer from four GABA pretreatment groups were randomly assigned to the same group’s polybags, each containing 6 fish. Afterward, the polybags were filled with an equal volume of pure oxygen until they expanded and were then promptly sealed with rubber bands. Every 12 packages were placed in one styrofoam box with ice bags inside and sealed with tape. To simulate transportation, the boxes were shaken up 5 times by a shaker (HY-5A, Changzhou, China) at 1 h intervals; each shaking time lasted for 5 min, and the shaking frequency was set at 100 rpm (revolutions per minute) [24,25]. No tawny puffer died during the experiment. Samples were collected immediately at the end of 0, 6, and 12 h of transportation. The total time of tawny puffer treated by GABA (immersion pretreatment + simulative transportation) is expressed as 3 d + 0 h, 3 d + 6 h, and 3 d + 12 h. The experimental design process is shown in Figure 1.

### 2.4. Sample Collection

After simulative transportation for 0, 6, and 12 h, the pH and DO were measured instantaneously with a water quality meter (HQ40d; Hach, Shanghai, China), and then water samples were collected to measure the TAN content according to the colorimetric phenol–hypochlorite method [26]. Tawny puffer were collected when transport finished and were anesthetized with 100 mg/L MS-222 (tricaine methanesulfonate, Sigma, St. Louis, MO, USA). Then, blood was sampled via cardiac puncture using a 1 mL medical syringe and was transferred to the anti-coagulating centrifuge tube. The collected blood was sat at 4 °C for 24 h and was then centrifuged at 1737× *g* for 10 min by high-speed refrigerated centrifuge (XiangYi Technologies, Changsha, China). The plasma was taken and stored at −20 °C to determine plasma biochemical indices. The liver was frozen in liquid nitrogen and stored at −20 °C. Before use, liver samples were homogenized in 0.86% physiological saline to prepare 10% homogenate, which was used to determine energy metabolism indicators and antioxidant indexes.

### 2.5. Plasma Biochemical Indices

The glucose (GLU) concentration in plasma was measured by the GLU oxidase method [27], and the experimental procedures were conducted strictly in accordance with the instructions of detection kits (Chinese Mindray company, Shenzhen, China). The method used to detect epinephrine (EPI) and cortisol (COR) in plasma was an enzyme-linked immunosorbent assay (ELISA). Briefly, 50 μL of sample or standard, along with 100 μL of horseradish peroxidase (HRP)-labeled detection antibody, were added to the antibody-coated 96-well plates, covered with microplate sealers, and then incubated for 60 min at 37 °C. After washing the plates 5 times, 50 μL of substrates A and B was added to each well and incubated at 37 °C for 15 min in the dark, followed by the addition of 50 μL of stop solution. The absorbance (OD value) was measured at 450 nm with a microplate reader (iMarkTM Microplate Reader, Bio-Rad, Hercules, CA, USA), and the sample concentration was calculated according to the curve equation. Commercial fish ELISA kits for EPI (ml260548) and COR (ml003467) were supplied by Shanghai Enzyme-linked Biotechnology (Shanghai, China).

### 2.6. Energy Metabolism Indicators

The content of total protein (TP), liver glycogen (LG), and lactic acid (LA) was quantified by the Coomassie brilliant blue method [28], anthrone colorimetric method [29], and lactate dehydrogenase method [30], respectively. The activities of ATPase (including Na^+^-K^+^-ATPase and Ca^2+^-Mg^2+^-ATPase) and lactate dehydrogenase (LDH) [31] were determined by the colorimetric method [32]. The activities of pyruvate kinase (PK) were measured by ultraviolet spectrophotometry [33]. The activities of phosphofructokinase (PFK) and citrate synthase (CS) were measured via an ELISA, for which the determination method was the same as that of EPI (ml260548) and COR (ml003467). The assay kits of TP (A045-2-2), LG (A043-1-1), LA (A019-2-1), ATPase (A016-2-2), LDH (A020-1-2), and PK (A076-1-1) were obtained from the Nanjing Jiancheng Bioengineering Institute (Nanjing, China); the commercial fish ELISA kits for PFK (ml270437) and CS (ml206952) were obtained from Shanghai Enzyme-linked Biotechnology (Shanghai, China). The OD value was read with a microplate reader and ultraviolet (UV) spectrophotometer (Shanghai Precision Scientific Instrument Co., Ltd., Shanghai, China), and then the contents of the corresponding indicators were calculated.

### 2.7. Antioxidant Indexes

The superoxide dismutase (SOD) activities were detected by the WST-1 (water-soluble tetrazolium salt) method [34], peroxidase (POD) activities were measured according to the method of He et al. [35], the total antioxidant capacity (T-AOC) was determined by the colorimetric method, and the contents of malondialdehyde (MDA) were determined by the TBARS (thiobarbituric acid reactive substance) assay [36]. The assay kits of SOD (A001-3-2), POD (A084-1-1), T-AOC (A084-1-1), and MDA (A003-1-2) were obtained from the Nanjing Jiancheng Bioengineering Institute (Nanjing, China), and the experimental procedures were carried out strictly according to the instructions. The OD values were measured with a UV spectrophotometer at 550 nm, 420 nm, 520 nm, and 532 nm, respectively.

## 3. Statistical Analyses

Data are presented as mean ± standard deviation (mean ± S.D.). The statistical analysis was carried out using the SPSS software (version 23.0 for Mac). An independent-sample *t*-test was used for intragroup comparison before and after stress, and a one-way ANOVA test was used for intergroup comparison at the same time point of stress. Significant differences were established at *p* < 0.05.

## 4. Results

### 4.1. Water Quality Parameters

As shown in Table 1, regarding the pH of seawater in polybags, after 6 h of transport, the pH in the 150 mg/L GABA treatment groups was significantly (*p* < 0.05) higher than the control group; after 12 h, the pH in the 50 and 150 mg/L GABA treatment groups was significantly (*p* < 0.05) higher than the control group. Regarding the DO of seawater in polybag, after 12 h of transport, the DO in the 50 and 150 mg/L GABA treatment groups was significantly (*p* < 0.05) higher than the control group. Regarding the TAN of seawater in polybag, after 6 and 12 h of transport, the TAN in the 150 mg/L GABA treatment groups was significantly (*p* < 0.05) lower than the control group.

### 4.2. Plasma Biochemical Indices

**Figure 2:**
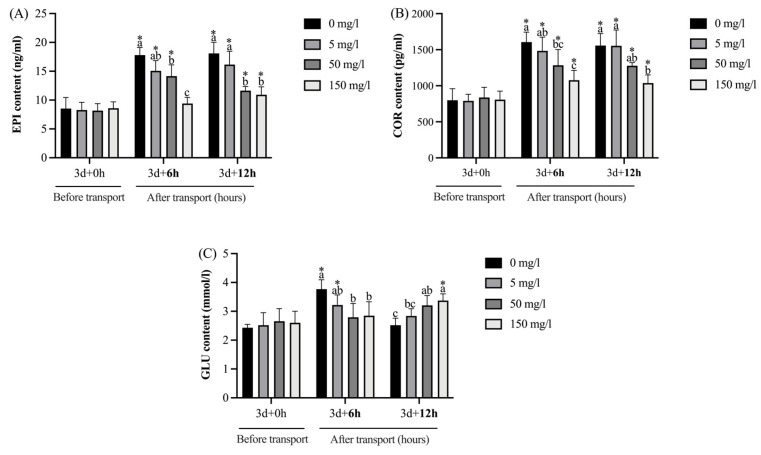
The effect of GABA treatment on the plasma biochemical indices of tawny puffer under transport stress. (**A**) Epinephrine (EPI) content; (**B**) cortisol (COR) content; (**C**) glucose (GLU) content. The values are expressed as the means ± SD (n = 6). Diverse letters above the column represent the significant differences (*p* < 0.05) in different groups at the same time point in Duncan’s test. Significant differences (*p* < 0.05) between values obtained before and after stress are marked by “*” in *t*-tests.

As shown in Figure 2, after GABA treatment for 3 days (0 h transport), no significant (*p* > 0.05) differences in the EPI, COR, and GLU contents of plasma were observed among the groups. Compared with pre-transportation, the EPI contents in all groups were significantly (*p* < 0.05) increased after undergoing transport, except the 150 mg/L GABA treatment group at transport for 6 h (*p* > 0.05). After 6 h of transport, the EPI contents in the 50 and 150 mg/L GABA treatment groups were significantly (*p* < 0.05) lower than the control group; after 12 h, the EPI contents in the 50 and 150 mg/L GABA treatment groups were significantly (*p* < 0.05) lower than those of the other groups. The COR contents in all the groups were also significantly (*p* < 0.05) increased after undergoing transport. After 6 h of transport, the COR contents in the 50 and 150 mg/L GABA treatment groups were significantly (*p* < 0.05) lower than the control group; after 12 h, the COR contents in the 150 mg/L GABA treatment groups were significantly (*p* < 0.05) lower than the control and 5 mg/L GABA treatment groups. Compared with pre-transportation, the GLU contents in the control and 5 mg/L GABA treatment group were significantly (*p* < 0.05) increased at transport for 6 h and then they fell back to before transport levels at 12 h (*p* > 0.05); the GLU contents in the 150 mg/L GABA treatment group were significantly (*p* < 0.05) increased at transport for 12 h. Furthermore, after 6 h of transport, the GLU contents in the 50 and 150 mg/L GABA treatment groups were significantly (*p* < 0.05) lower than the control group; after 12 h, the GLU contents in the 50 and 150 mg/L GABA treatment groups were significantly (*p* < 0.05) higher than the control group.

### 4.3. Energy Metabolism Indicators

**Figure 3:**
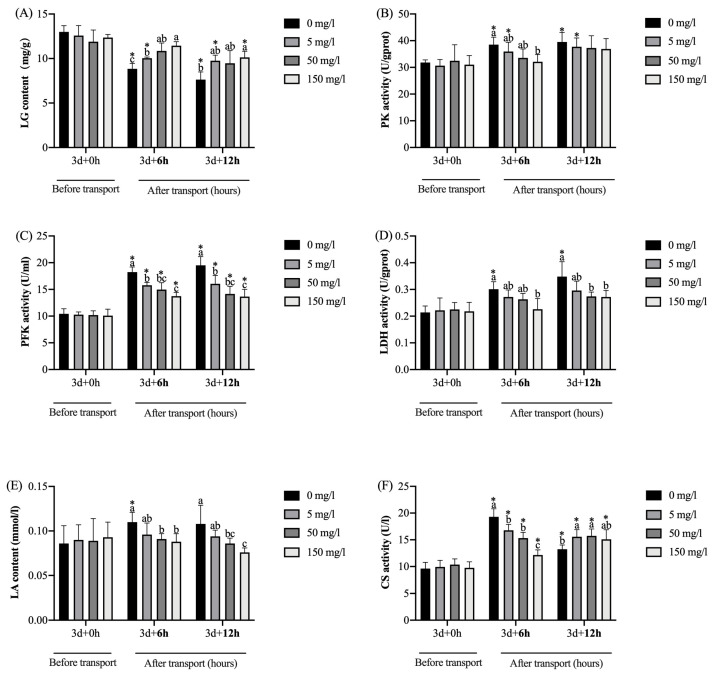
The effect of GABA treatment on the energy metabolism indicators in the liver of tawny puffer under transport stress. (**A**) Liver glycogen (LG) content; (**B**) pyruvate kinase (PK) activity; (**C**) phosphofructokinase (PFK) activity; (**D**) lactate dehydrogenase (LDH) activity; (**E**) lactic acid (LA) content; (**F**) citrate synthase (CS) activity. The values are expressed as the means ± SD (n = 6). Diverse letters above the column represent the significant differences (*p* < 0.05) in different groups at the same time point in Duncan’s test. Significant differences (*p* < 0.05) between values obtained before and after stress are marked by “*” in *t*-tests.

As shown in Figure 3, after GABA treatment for 3 days (0 h transport), no significant (*p* > 0.05) differences in the contents of LG and LA and the activities of LDH, PK, PFK, and CS in the livers were observed among the groups. Compared with pre-transportation, the LG contents in the control and 5 mg/L GABA treatment group were significantly (*p* < 0.05) decreased at transport for 6 h; the LG contents in the control, 5 mg/L, and 150 mg/L GABA treatment groups were significantly (*p* < 0.05) decreased at 12 h. Furthermore, after 6 h of transport, the LG contents in all the GABA treatment groups were significantly (*p* < 0.05) higher than the control group; after 12 h, the LG contents in the 150 mg/L GABA treatment groups were significantly (*p* < 0.05) higher than the control group. Compared with pre-transportation, the PK activities in the control and 5 mg/L GABA treatment groups were significantly (*p* < 0.05) increased after undergoing transport. After 6 h of transport, the PK activities in the 150 mg/L GABA treatment groups were significantly (*p* < 0.05) lower than in the control group. The PFK activities in all the groups were significantly (*p* < 0.05) increased after undergoing transport. After 6 and 12 h of transport, the PK activities in all the GABA treatment groups were significantly (*p* < 0.05) lower than the control group. Compared with pre-transportation, the LDH activities in the control group were significantly (*p* < 0.05) increased after undergoing transport. After 6 h of transport, the LDH activities in the 150 mg/L GABA treatment groups were significantly (*p* < 0.05) lower than the control group; after 12 h, the LDH activities in the 50 and 150 mg/L GABA treatment groups were significantly (*p* < 0.05) lower than the control group. Compared with pre-transportation, the LA contents in the control group were significantly (*p* < 0.05) increased at transport for 6 h. After 6 and 12 h of transport, the LA contents in the 50 and 150 mg/L GABA treatment groups were significantly (*p* < 0.05) lower than the control group. The CS activities in all the groups were significantly (*p* < 0.05) increased after undergoing transport, and the control group displayed a tendency to first rise and then decline with the extension of transport time. After 6 h of transport, the CS activities in all the GABA treatment groups were significantly (*p* < 0.05) lower than the control group; after 12 h, the CS activities in the 5 and 50 GABA treatment groups were significantly (*p* < 0.05) higher than the control groups.

**Figure 4:**
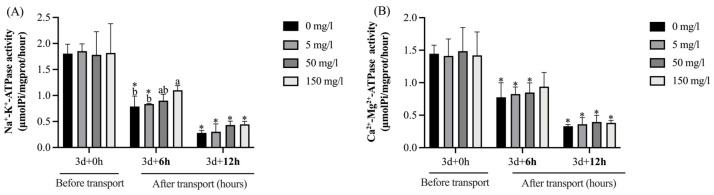
The effect of GABA treatment on the ATPase activities in the liver of tawny puffer under transport stress. (**A**) Na^+^-K^+^-ATPase activities; (**B**) Ca^2+^-Mg^2+^-ATPase activities. The values are expressed as the means ± SD (n = 6). Diverse letters above the column represent the significant differences (*p* < 0.05) in different groups at the same time point in Duncan’s test. Significant differences (*p* < 0.05) between values obtained before and after stress are marked by “*” in *t*-tests.

As shown in Figure 4, after GABA treatment for 3 days (0 h transport), no significant (*p* > 0.05) differences in the activities of Na^+^-K^+^-ATPase and Ca^2+^-Mg^2+^-ATPase in the livers were observed among the groups. Compared with pre-transportation, the Na^+^-K^+^-ATPase activities in the control and 5 mg/L GABA treatment group were significantly (*p* < 0.05) decreased at transport for 6 h, and the Na^+^-K^+^-ATPase activities in all the groups were significantly (*p* < 0.05) decreased at 12 h. After 6 h of transport, the Na^+^-K^+^-ATPase activities in the 150 mg/L GABA treatment group were significantly (*p* < 0.05) higher than the control and 5 mg/L GABA treatment group. The Ca^2+^-Mg^2+^-ATPase activities in all groups were significantly (*p* < 0.05) decreased after undergoing transport, except the 150 mg/L GABA treatment group at transport for 6 h (*p* > 0.05). After 6 and 12 h of transport, no significant (*p* > 0.05) differences were observed among each group.

### 4.4. Antioxidant Indexes

**Figure 5:**
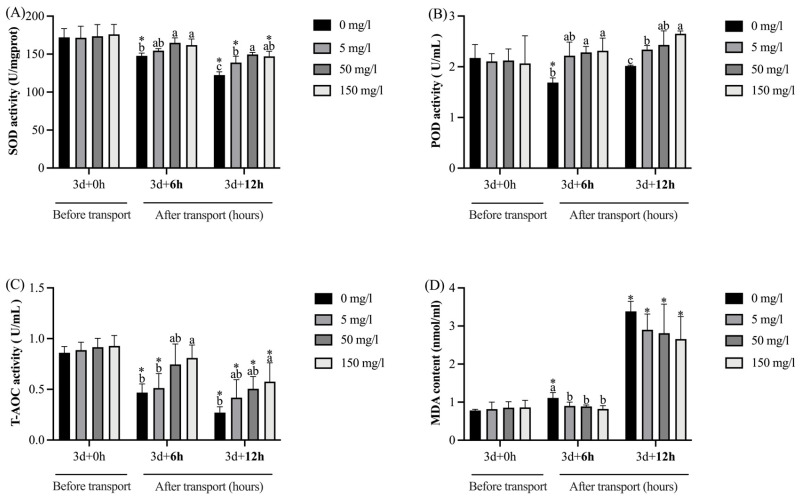
The effect of GABA treatment on the antioxidant indexes in the liver of tawny puffer under transport stress. (**A**) Superoxide dismutase (SOD) activity; (**B**) peroxidase (POD) activity; (**C**) total antioxidant capacity (T-AOC); (**D**) malondialdehyde (MDA) content. The values are expressed as the means ± SD (n = 6). Diverse letters above the column represent the significant differences (*p* < 0.05) in different groups at the same time point in Duncan’s test. Significant differences (*p* < 0.05) between values obtained before and after stress are marked by “*” in *t*-tests.

As shown in Figure 5, after GABA treatment for 3 days (0 h transport), no significant (*p* > 0.05) differences in the activities of SOD, POD, and T-AOC and the contents of MDA in the livers were observed among the groups. Compared with pre-transportation, the SOD activities in the control group were significantly (*p* < 0.05) decreased at transport for 6 h; the SOD activities in the control, 5, and 150 mg/L GABA treatment groups were significantly (*p* < 0.05) decreased at 12 h. Furthermore, after 6 h of transport, the SOD activities in the 50 and 150 mg/L GABA treatment groups were significantly (*p* < 0.05) higher than the control group; after 12 h, the SOD activities in all the GABA treatment groups were significantly (*p* < 0.05) higher than the control group. Compared with pre-transportation, the POD activities in the control group were significantly (*p* < 0.05) decreased at transport for 6 h. Furthermore, after 6 h of transport, the POD activities in the 50 and 150 mg/L GABA treatment groups were significantly (*p* < 0.05) higher than the control group; after 12 h, the POD activities in all the GABA treatment groups were significantly (*p* < 0.05) higher than the control group. Compared with pre-transportation, the T-AOC levels in the control and 5 mg/L GABA treatment groups were significantly (*p* < 0.05) decreased at transport for 6 h; the T-AOC levels in all the groups were significantly (*p* < 0.05) decreased at 12 h. After 6 h of transport, the T-AOC levels in the 150 mg/L GABA treatment groups were significantly (*p* < 0.05) higher than the control and 5 mg/L groups; after 12 h, the T-AOC levels in the 150 mg/L GABA treatment groups were significantly (*p* < 0.05) higher than the control group. Compared with pre-transportation, the MDA contents in the control groups were significantly (*p* < 0.05) increased at transport for 6 h; the MDA contents in all the groups were significantly (*p* < 0.05) increased at 12 h. After 6 h of transport, the MDA contents in all the GABA treatment groups were significantly (*p* < 0.05) lower than the control group; after 12 h, no significant (*p* > 0.05) differences were observed among each group.

## 5. Discussion

In this study, with prolonged transportation time, the pH and DO levels tended to decrease, and the TAN tended to increase in all groups. The study of silver catfish (*Rhamdia quelen*) showed the same variation trend in terms of pH, DO, and TAN levels with this study after live transport [37]. Relevant studies have manifested that transport enhances metabolic activity and increases the respiratory frequency (RF) and oxygen consumption rate (OCR) of fish, which leads to the consumption of DO in transport water [38]. Meanwhile, increased metabolism results in the release of high levels of metabolites, including carbon dioxide and non-ionic ammonia, causing a reduction in pH levels and an elevation in ammonia nitrogen in transport water [39]. Prolonged exposure to ammonia nitrogen inhibits the excretion of ammonia in fish, leading to diminished oxygen-carrying capacity in the blood and further elevated levels of ammonia nitrogen in water [40,41], ultimately inducing pathological changes in the gill, liver, and kidney, even death of fish [42,43]. Elevated levels of CO_2_ in blood have been shown to induce acidification, which also decreases the oxygen-carrying capacity and hemoglobin’s affinity for oxygen [44]. In brief, a combination of deteriorating water parameters affects fish physiology and health negatively. Noteworthy, in this study, 50 or 150 mg/L GABA treatment inhibited pH, DO decrease, and TAN increase in water after 6 and 12 h of transport compared with the control group. Water quality can intuitively reflect the metabolic status of fish during transportation, indicating that GABA treatment reduced the metabolic level of fish and prevented the deterioration of water quality during transportation. Similar outcomes were noted in *Cyprinus carpio* following transportation in water supplemented with GABA [21]. This highlights the potential benefits of using GABA as a metabolic inhibitor to prevent water quality degradation in live transport.

The primary stress response of fish starts with the neuroendocrine reaction, which activates the SC axis and HPI axis and triggers the release of EPI and COR, respectively. EPI primarily provides energy in the short term by promoting glycogenolysis; COR provides energy in the long term by promoting the gluconeogenesis of GLU and the catabolism of proteins and fats [45]. The contents of EPI and COR have been seen as common indexes to reflect the degree of stress in organisms [46]. In the current study, the content of EPI and COR tended to increase after 6 and 12 h of transport. However, 50 or 150 mg/L GABA supplementation inhibited the increase in EPI and COR after 6 and 12 h of transport compared with the control group, which reflected the anti-stress effect of GABA. The study of *Carassius auratus* [20] and *Cyprinus carpio* [21] also showed that GABA addition in water suppressed the rise of EPI and COR effectively during transportation. On the one hand, GABA is a commonly used sedative that may effectively mitigate the activation of the HPI axis in fish [47], thereby preventing their response to further stressors and inducing a cascade of physiological and metabolic adjustments aimed at preserving homeostasis [48]; on the other hand, GABA is an inhibitory neurotransmitter in the nervous system, which functions via GABAa, GABAb, and GABAc receptors. When GABA acts at GABAa receptors, the enhanced permeability of nerve cell membranes to chloride ions results in their influx into the cell, thereby inducing membrane potential elevation and hyperpolarization, ultimately culminating in diminished excitation [49]. This might be the potential mechanism which meant that GABA suppressed the increase in EPI and COR effectively during tawny puffer transportation in the present study. With the extension of transport time, the GLU content in the control groups was significantly increased at 6 h and then fell back to the levels observed before transport at 12 h. This phenomenon could be attributed to the rapid secretion of EPI under transport stress, leading to glycogenolysis, increasing the plasma GLU level significantly, and then GLU was continuously consumed after 12 h of transport. Plasma GLU is a critical stress level indicator [50], indicating that the control group experienced stress after 6 h of transport. However, the GLU content in the 50 and 150 mg/L GABA treatment groups increased slowly and continuously with the extension of transportation. GLU homeostasis can reduce the production and accumulation of toxic substances, which is extremely important for fish health. Numerous prior studies have documented the beneficial impact of GABA in the regulation of GLU metabolism and the maintenance of blood GLU homeostasis [51,52], consistent with this research. Overall, GABA treatment can alleviate transport stress in tawny puffer by suppressing the increase in EPI and COR and maintaining GLU homeostasis.

Fish experience hypoxia during transportation due to the continuous decrease in DO in the water and the decline in the oxygen transport capacity caused by the accumulation of CO_2_ and ammonia nitrogen. The typical response to hypoxia in fish is altering energy metabolism mode [53], and the liver is a major organ for energy metabolism and stores large amounts of glycogen. When fish encounter hypoxia, liver glycogen is broken down into glucose, and glucose is an indispensable substrate for glycolysis. Under the catalysis of PFK and PK, glycolysis occurs and produces a small amount of ATP. However, the efficiency of glycolysis in generating energy is limited and accompanied by the production of a large amount of LA, which induces a series of negative effects on energy-consuming organisms [54]. LA is the terminal product of glycolysis, and the large amount of LA that accumulates in the liver can be converted into GLU by gluconeogenesis or into pyruvate and enter the tricarboxylic acid (TCA) cycle, and LDH catalyzes the interconversion of pyruvate and LA [55]. The PFK, PK, and LDH activities reflect anaerobic metabolism’s ability in organisms [56]. The TCA cycle is an essential segment for aerobic metabolism and can produce a large amount of ATP. CS is the rate-limiting enzyme of the TCA cycle, whose activity is frequently used to estimate aerobic capacity in animals [57]. In this study, the LG content in the control group gradually decreased with the prolongation of transportation; however, GABA treatment could inhibit the decline of LG. From the results of anaerobic metabolism-related enzymes, after 6 and 12 h of transport, the PK, PFK, and LDH activities in the control group were higher than those in the GABA treatment group; the LA content showed the same change in tendency. This indicated that the control group improved and maintained anaerobic metabolism levels under transport stress, producing a large amount of LA. This phenomenon could be attributed to the intense activity of fish caused by transportation stress [58] and the hypoxia caused by the continuous deterioration of water quality in the later stages of transportation. Meanwhile, GABA treatment could suppress the upregulation of anaerobic metabolism levels and inhibit LA accumulation. According to relevant research, GABA has been found to inhibit the excitability of neurons that increase anaerobic pathways [59]. This may be an important pathway for GABA to regulate metabolism under transport stress. From the results of aerobic metabolic enzymes, compared with before transport, the CS activities of the control groups significantly increased at transport for 6 h and then fell back at 12 h. This indicated that the tawny puffer of the control group enhanced aerobic and anaerobic metabolism for adaptive adjustment after 6 h of transport; after 12 h, aerobic metabolism was inhibited and mainly relied on anaerobic metabolism for energy supply. However, anaerobic metabolism cannot compensate for the ATP deficit caused by aerobic metabolism inhibition, and continuous hypoxic disrupts energy homeostasis in vivo. It is worth noting that the CS activities in the GABA treatment groups were lower than the control group after 6 h of transport and higher than the control group after 12 h. This indicated that the tawny puffer treated by GABA maintained a stable level of aerobic metabolism during transportation, which is consistent with the research results of Zhang et al. and Varghese et al. [19,60]. This phenomenon could potentially be attributed to the tawny puffer of the GABA treatment group exhibiting better water quality and a lower degree of stress following transportation, thereby enabling sustained aerobic metabolism. This discovery suggests that GABA regulates energy metabolism in tawny puffer experiencing transport stress by suppressing the upregulation of anaerobic metabolism and maintaining aerobic metabolism levels.

Na^+^-K^+^-ATPase and Ca^2+^-Mg^2+^-ATPase are widely distributed biofilm enzyme systems in the organism which use energy from ATP hydrolysis to maintain the balance of the ion concentration between the inside and outside of the cells [61]. In the present study, after 6 h of transport, the Na^+^-K^+^-ATPase and Ca^2+^-Mg^2+^-ATPase activities in the liver of the control group were significantly decreased compared with pre-transportation. A reduction in the activities of Na^+^-K^+^-ATPase results in elevated intracellular Na^+^ levels, leading to cellular edema. Similarly, a decrease in Ca^2+^-Mg^2+^-ATPase activities leads to intracellular Ca^2+^ overload, which damages the mitochondrial structure and function, disrupts oxidative phosphorylation processes, and ultimately hinders ATP synthesis. This result demonstrated the adverse effects of transportation stress on fish. One possible explanation is that stress-induced metabolic processes generate an increase in reactive oxygen species (ROS) production [10], which may result in inhibited activities of ATPase [62]. Notably, after 6 h of transport, the Na^+^-K^+^-ATPase of the 50 and 150 GABA treatment groups was significantly higher than those in the control group, which illustrated the advantageous effects of GABA treatment in fish transportation.

Stress-induced accumulation of ROS can lead to oxidative damage in organisms [10]. Antioxidant enzymes play a crucial role in maintaining oxidation–reduction homeostasis by modulating the production and scavenging of ROS [63]; thus, the antioxidant enzyme activities serve as a valuable metric for gauging the antioxidant potential of the organism [64]. The principal antioxidant enzyme includes SOD and POD. Superoxide anion-free radicals are dismutated by SOD into hydrogen peroxide (H_2_O_2_), and H_2_O_2_ is removed by POD to generate H_2_O. T-AOC reflects the overall antioxidant capability of organisms. MDA is a lipid peroxidation product whose content is proportional to the extent of lipid oxidative damage. In the present study, at transport for 6 and 12 h, the SOD and POD activities and T-AOC levels of the control group were lower, and MDA was higher than before transport. It is possible that the tawny puffer of the control group counteracted the production of ROS by consuming antioxidant enzymes, but lipid oxidative damage still cannot be prevented. Noteworthy, after 6 and 12 h of transport, the SOD and POD activities and T-AOC levels of the GABA treatment groups were higher, and MDA was lower than those in the control group. Similar results have been reported in the study of white shrimps (*Litopenaeus vannamei*) and grass carp (*Ctenopharyngodon idellus*) [65,66]. This phenomenon can be attributed to GABA’s function of improving antioxidant capacity and reducing oxidative damage. The role of GABA in the oxidation–reduction system primarily involves its interaction with ROS intermediates [67]; the metabolic process of GABA contributes to the regulation of and reduction in ROS accumulation [68]. In addition, GABA has been confirmed to trap MDA indirectly or directly, thereby protecting the organism from oxidative damage [69]. As previously mentioned, the higher Na^+^-K^+^-ATPase activities observed in the 50 and 150 GABA treatment groups after 6 h of transport may be attributed to the antioxidant properties of GABA. These results preliminarily illustrated the mechanism of GABA as an antioxidant to alleviate transport stress.

## 6. Conclusions

The present study revealed that immersing tawny puffer in GABA-containing seawater for 3 days, followed by simulative transportation using seawater with the same GABA levels, effectively alleviated transport stress, especially in 50 and 150 mg/L GABA concentrations. GABA exerted anti-stress functions by avoiding the deterioration of transport water quality, inhibiting the elevation in stress-related biochemical indicators, regulating energy metabolism, and mitigating oxidative damage. Therefore, GABA can be used as an effective additive for tawny puffer live transport. This study serves as a reference for future research on reducing transportation stress in aquatic animals and investigating the anti-stress mechanisms of GABA.

## Figures and Tables

**Table 1:** The effect of GABA treatment on the pH, dissolved oxygen (DO), and total ammonium nitrogen (TAN) of water after tawny puffer transport.

Indexes	0 mg/L GABA	5 mg/L GABA	50 mg/L GABA	150 mg/L GABA
3 d + 0 h				
pH	7.48 ± 0.02	7.55 ± 0.18	7.46 ± 0.13	7.49 ± 0.28
DO (mg/L)	8.77 ± 0.15	8.57 ± 0.25	8.63 ± 0.25	8.60 ± 0.44
TAN (mg/L)	0.27 ± 0.06	0.24 ± 0.09	0.27 ± 0.03	0.26 ± 0.04
3 d + 6 h				
pH	7.08 ± 0.03 ^b^	7.16 ± 0.06 ^ab^	7.20 ± 0.06 ^ab^	7.26 ± 0.11 ^a^
DO (mg/L)	6.47 ± 0.15	6.73 ± 0.15	6.80 ± 0.26	6.77 ± 0.25
TAN (mg/L)	7.31 ± 0.22 ^a^	6.79 ± 0.96 ^ab^	6.44 ± 1.09 ^ab^	5.97 ± 0.32 ^b^
3 d + 12 h				
pH	6.62 ± 0.19 ^b^	6.75 ± 0.23 ^ab^	7.08 ± 0.03 ^a^	7.05 ± 0.02 ^a^
DO (mg/L)	3.37 ± 0.15 ^c^	3.60 ± 0.20 ^bc^	3.87 ± 0.21 ^ab^	4.23 ± 0.31 ^a^
TAN (mg/L)	9.79 ± 1.24 ^a^	9.00 ± 0.75 ^ab^	8.49 ± 0.97 ^ab^	7.95 ± 0.44 ^b^

Note: The values are expressed as the means ± SD (n = 3). Means in the same row with different superscript letters are significantly different (*p* < 0.05); conversely, means in the same row without superscript letters indicate no significant difference (*p* > 0.05).

## Data Availability

The datasets generated during and/or analyzed during the current study are available from the corresponding author upon reasonable request.
